# Signatures

**Published:** 1885-09

**Authors:** 


### Signatures.

None but publishers have any adequate idea of the number of persons who cannot, or at least fail to write their names with sufficient distinctness to be deciphered. A letter can be made out, be it written ever so badly, because aid is given by the connection; but conjecture can do no good in making out a name which is disfigured by innumerable and senseless flourishes, which bear no similitude to any letter in the English or any other language; then as to the minor letters, the *e*'s, *i*'s, *n*'s, *m*'s, *u*'s, *v*'s and *w*'s, they appear nothing more than a long line of zigzag, or a Virginia worm fence turned on its side. An incalculable amount of trouble, uncertainty, and loss of time, to say nothing of cost of postages, envelopes, and paper, would be prevented if every person would write their own name at leisure, without any flourishes, with the utmost distinctness, every letter fully, clearly, and plainly formed; or if an answer is desired, to inclose a post-paid envelope with at least the name of the state, town, and county plainly written; then, should it reach the post-office, the poot-master might possibly guess at the writer. One additional advantage of this method would be that the person who is desired to answer the hieroglyphic sheet would not be meanly taxed with return postage and envelope. A small consideration, it is true; but it involves a principle of honesty and morality which every high-minded man feels bound to respect.

